# Reconciling evolution: evidence from a biology and theology course

**DOI:** 10.1186/s12052-020-00133-9

**Published:** 2020-09-02

**Authors:** Ethan R. Tolman, Daniel G. Ferguson, Mark Mann, April Maskiewicz Cordero, Jamie L. Jensen

**Affiliations:** 1 Department of Plant and Wildlife Sciences, Brigham Young University, 4125 LSB, Provo, UT 84602 USA; 2 Department of Biology, Brigham Young University, 4102 LSB, Provo, UT 84602 USA; 3 School of Theology and Christian Ministry, Point Loma Nazarene University, 3900 Lomaland Dr., San Diego, CA 92106 USA; 4 Department of Biology, Point Loma Nazarene University, 3900 Lomaland Dr., San Diego, 92106 USA

**Keywords:** Evolution, Higher education, Theology, Biology, Acceptance

## Abstract

**Background:**

Many individuals reject evolutionary theory due to a perceived conflict with their religious beliefs. To bridge this gap, educators have attempted different approaches including approaching evolution rejection as a consequence of deficit thinking and teaching students the nature of science (including the scientific process and peer review process as well as questions that science can and cannot answer).Teaching the nature of science has shown promising gains in the acceptance of evolution, although acceptance rates remain low. We propose a further approach: the use of a reconciliatory model designed to help students accept evolution within the framework of their religious beliefs. We tested this approach in both biology and theology classrooms at a Nazarene-affiliated university. Both professors approached the subject in a reconciliatory fashion.

**Results:**

This study found that by utilizing a reconciliatory approach, the students in both classrooms saw significant gains in evolution acceptance, with gains being greatest in the biology classroom. In addition, we saw no decrease in student religiosity.

**Conclusions:**

Implications of this are discussed. The results of this study confirm the effectiveness of a reconciliatory model, which opens several avenues for further research.

## Background

Overall acceptance of evolutionary theory in the United States is exceptionally low. A 2019 Gallup poll found that 40% of Americans still hold creationist views (Brenan 2019). Additionally, polling in Europe conducted by the Pew Research Center (Science and religion in Central and Eastern Europe 2017) found that 29% of Greeks, 26% of Russians and 23% of Poles believe humans “existed in (the) present state since (the) beginning of time.”

Experts have argued that rejection of evolutionary theory in the United States is strongly correlated with religious affiliation. Forty-two percent of Christians say humans have remained the same throughout time compared to 18% of the overall population (Exploring Different Ways of Asking About Evolution 2019). This large rejection of evolution amongst American Christians can be attributed to a number of things including a belief that God is an ally or co-pilot of America (Reinforced Theistic Destiny 2012), epistemology of appealing to authority (Borgerding et al. 2017; Peterson 2019), or socio-cultural factors including ingroup-outgroup anxieties and existential fear. Not surprisingly, acceptance varies by denomination. For instance, white Evangelical Protestants have the highest percentage of people who reject human evolution, with a reported 38% believing humans have always existed in their present form (Exploring Different Ways of Asking about Evolution 2019). Science educators have tried several approaches to increase the acceptance of evolutionary theory as a foundational theory in biology. In this paper we discuss some of these methods and then examine the effectiveness of a reconciliatory approach in both a college level theology and biology class.

## Deficit thinking

The deficit model is a commonly held perspective where instructors assume that evolution rejection is due to either a deficit in knowledge of evolutionary theory, or a deficit in reasoning ability (Baum 2009; Lawson and Weser 1990), and as such educators focus on teaching more facts. Data in the literature conflict about whether greater knowledge of the facts of evolution is correlated with acceptance, with some studies showing a connection between evolution acceptance and knowledge (Dunk et al. 2017; Johnson and Peeples 1987; Rutledge and Warden 2000; Weisberg et al. 2018) and others finding no correlation (Bishop and Anderson 1990; Chinsamy 2008; Mead et al. 2017). These mixed findings suggest that the relationship between knowledge and acceptance is promising, but likely more complicated than just delivering facts (Rosengren et al. 2012). In addition, a persistent narrative by many evolution-accepting members of the public claims that those who reject evolution are merely irrational or uneducated (Pobiner 2016). These types of inaccurate accusations have the potential to activate defense mechanisms that can alter one’s perception of ideas (Vailiant et al. 2001) and thus potentially negatively affect evolution acceptance.

While the literature shows a plausible connection between a lack of knowledge of the facts of evolution, no research supports the claim that those who do not accept evolution have a deficit in their reasoning ability. In fact, recent research suggests the opposite; that no relationship exists between scientific reasoning ability and evolution acceptance (Jensen et al. 2019). Despite a lack of data, this view is held by outspoken and prominent biologists, such as Dawkins (2008, 2019), who advocate that those who do not believe in evolution are deficient in one or more areas. Research has shown this misinformation and flawed reasoning approach to be harmful to student learning (Barnes and Brownell 2016; Barnes et al. 2017). It is our experience that a deficit approach not only fails to increase acceptance of evolution among students, but it can push them further away from accepting evolutionary theory.

## The influence of a role model

Several researchers have experimentally found that exposing students to role models who hold a belief in God and accept evolutionary theory can positively impact students’ acceptance of evolution (Barnes and Brownell 2017; Holt et al. 2018). A role model could be the professor teaching the course or the professor could highlight other scientific figures who accept both religion and evolution such as Collins (2008) or Stephen Jay Gould (Masci 2009). Lastly, one could use the teachings of religious figures such as Father Coyne (2020) and Pope Francis (Tharoor 2020) to give students a pathway towards reconciling evolution and faith. For this current study, the instructors themselves shared the religious cultures of a large portion of the student body and served as an ideal role model.

## Teaching the nature of science

Another approach to increase evolution acceptance involves ensuring that students have a very clear understanding of the nature of science (e.g. (Dunk et al. 2017)). The National Science Teachers’ Association states that

Although no single universal step-by-step scientific method captures the complexity of doing science, a number of shared values and perspectives characterize a scientific approach to understanding nature. Among these are a demand for naturalistic explanations supported by empirical evidence that are, at least in principle, testable against the natural world. Other shared elements include observations, rational argument, inference, skepticism, peer review, and reproducibility of the work. This characteristic of science is also a component of the idea that “science is a way of knowing” as distinguished from other ways of knowing (National Science Teachers Association (NSTA) 2020).

Research has shown that emphasizing the nature of science can lead to gains in acceptance. For example, Cofré et al. (2017) found that gains in the understanding of NOS amongst teachers participating in a workshop was correlated with an increase of understanding and acceptance of evolutionary theory over the course of the workshop. A multifactorial analysis by Dunk et al. (2017) suggested that out of factors including evolutionary content knowledge, religiosity, epistemological sophistication, and an understanding of the nature of science, the latter was the most important factor associated with acceptance of evolution amongst college students, explaining four times more variation than evolutionary content knowledge. Nelson et al. (2019) argue that teaching the nature of science may be the most effective tool to increasing understanding of evolution. Additional work (Rutledge and Warden 2000; Rosengren et al. 2012; Akyol et al. 2010; Cavallo 2008; Lombrozo et al. 2008) has also found a connection between an understanding of NOS and acceptance of evolutionary theory. While varying instruments for measuring both NOS and evolution acceptance make the definitive claim of a relationship tenuous, research thus far strongly suggests that understanding the nature of science can be influential in promoting student acceptance. However, while NOS is an important building block for acceptance of evolution, we propose that amongst religious individuals, more may be required to increase acceptance.

## The influence of religiosity

Studies have shown that religiosity is an important predictor of acceptance (Jensen et al. 2019; Baker 2013) independent of the other factors listed above. Because of this, addressing perceived religious conflicts in addition to teaching the nature of science could potentially be an important factor in improving overall acceptance of evolutionary theory. Winslow’s (2011) seminal interview study explored 15 undergraduate Christian science students seeking to uncover their views on creationism and evolution, and their perspectives about the reconciliation of science and religion (Winslow et al. 2011). His interviews showed that accepting evolution was much more complicated than just considering the evidence. Students had to navigate a network of affective and contextual factors that influenced their worldview and position towards evolution. “Christian biology-related majors at a Christian university were able to retain a belief in God and accept evolution, thus achieving a measure of reconciliation between evolution and their personal religious beliefs” (p. 1047). This current study builds on Winslow’s research by trying to better understand how classroom instruction can promote reconciliation.

Brownell and colleagues compiled a list of factors that positively influence acceptance of evolution by reducing students’ belief in conflict between science and religion (Barnes and Brownell 2017). Their instructional model, Religious Cultural Competence in Evolution Education (ReCCEE), specifically outlines a set of practices to improve the inclusiveness of instruction and respect the cultural differences for instructors who may not necessarily share the culture of their students. In this current study, both instructors implement one of the suggested ReCCEE practices, highlighting potential compatibility, and from this point forward will be labeled as a reconciliatory approach. Lindsay et al. (2019) found that a reconciliatory approach accompanied by NOS instruction led to gains in evolution acceptance among undergraduates of several different Christian denominations. Surprisingly, even when instructors know that this approach can be effective, many still take an antagonistic view toward religion when teaching evolution (Barnes and Brownell 2016). We propose that, at least among highly religious individuals, using a reconciliatory approach that shows students how faith and evolution are not mutually exclusive, along with teaching the facts and the nature of science, can promote gains in evolution acceptance without a decrease in religiosity. It is important to emphasize that this approach is intended to increase scientific acceptance but not at the expense of religious belief.

## The reconciliatory approach

A reconciliatory approach emphasizes the compatible nature of evolution with most Judeo-Christian beliefs (see 28–31, for examples). It is an approach that seeks to minimize the dichotomy between faith and science, which is far more likely to be a result of what particular religious communities affirm, rather than the institutional commitments and ethos of their affiliate colleges and universities (Rosengren et al. 2012). Prior research gives reason to believe that an intervention solely reliant on reducing conflict can be effective (e.g. Lindsay et al. 2019; Manwaring et al. 2015). Given the nature of a reconciliatory approach, we hypothesize that such a method would be effective in producing increased acceptance of evolutionary theory in both biology and theology classrooms. A research poll found that Americans who reject human evolution dropped from 31 to 18% when respondents were able to specify that they believed God had a role to play in the evolutionary process (Exploring Different Ways of Asking About Evolution 2019), and Truong et al. (2018) found that just 6 min of instruction with the goal of reducing the conflict students perceived between evolution and religion led to a reduction of perceived conflict in 80% of sampled students.

A reconciliatory approach has much promise for promoting evolution acceptance but more empirical data is needed. In this study, we describe two reconciliatory approaches in two diverse courses for the purpose of showing their effectiveness on improving evolution acceptance: one in a biology classroom where the instructor is a scientific authority, and the other in a theology classroom where the instructor is a religious authority. We also describe the results of the reconciliatory approach on student religiosity.

## Materials and methods

### Population

In order to test effectiveness of a reconciliatory approach in each course, we recruited student participation from an introductory biology class (N = 53) and from three theology classes (N = 71) at a Nazarene-affiliated private university. The Biology class was taught by a tenured biology professor while the theology class was taught by a tenured theology professor. Students enrolled in the introductory biology course were mostly traditional freshman science majors. The theology class is an upper-division general education course focusing on Christian history and beliefs and generally includes non-theology major juniors and seniors.

### Intervention

In the biology course, the mechanisms of evolution and associated case studies are presented over an eight-week period from small-scale evolution through human evolution. In addition to teaching students about evolution, the professor’s goal was to expose students to multiple perspectives on the relationship between the Bible and creation. Brief reconciliation activities were infused both in the classroom and as homework (see Table 1), and the instructor shared her own personal story of reconciling evolution and faith providing students a role model to follow (for more detail on the lesson, please refer to the Wesleyan lesson plans at RecoEvo website (2020)).

**Table 1:** Biology reconciliation lesson plans

Biology Course—Reconciliation activities (Evolution content is primary focus throughout the implementation of these additional activities during the 8 weeks of instruction.)
1. Pre-Class Assignment(s): Students read Chapters 1–6, 8, and 10 of *Origins*, by Haarsma and Haarsma
2. Activity 1—Hearing from students about evolution and faith: Students write a conversation between a Christian and non-Christian about evolution. This allows students to feel that their “side” is heard
3. Activity 2—Creating space to learn about evolution: Students respond to “clicker” questions about their knowledge about evolution
4.Activity 3—Peer discussion, on Discussion Board. Provides students time to articulate their thoughts about evolution with their peers
5. Activity 4—Peer discussion, face-to-face in small groups. Provides space to dialogue about difficult issues at the intersection between evolution and faith
6. Activity 5—Analyzing how we treat others: Students read about hospitable dialogue vs tolerance, analyze their initial conversation from Activity 1, and then engage in a real dialogue with a person who holds an alternate vie
7. End of course Essay—students write about their position towards evolution and any changes that occur

In the theology course, evolution content is not addressed explicitly at any length. Instead, over two course sessions (total ~ 120 min), the professor focuses on biblical and theological issues relating to contemporary conflicts around evolutionary science. In particular, students are introduced to ways of reading the Bible that encourages the integration of Christian faith with science. In the first session, students read and then discuss *A Teacher’s Guide to Understanding Scripture* by Sam Powell, which enunciates a Wesleyan view of scripture.^1^ According to this view, the Bible is inspired by God, revelatory of God’s truths, and therefore authoritative for Christians in those areas in which it is concerned—that is, Christian faith and practice, and not, for instance, the age of the universe.^2^ In the second session, students are introduced to a variety of models for integrating science and faith, and read brief personal stories by persons who have come to affirm such integration. Finally, students address biblical texts often cited as conflicting with science (such as the creation story in Genesis 1 and flood story in Genesis 6), and are shown how these passages can accommodate contemporary science while maintaining deep theological meaning and value for considerations of Christian faith and practice.

### Outcome measures

To measure student acceptance of evolution, we administered the Generalized Acceptance of EvolutioN Evaluation (GAENE; (Smith et al. 2016)) prior to the intervention and then at the end of the evolution module via an online surveying platform. Validity and reliability of the instrument is detailed in Smith et al. (2016), Cronbach’s alpha = .941. Students were given a small amount of course credit for completing the surveys. The GAENE contains 13-items, each measured on a 5-point Likert scale. The total score is the sum of responses on all 13 items. The change in acceptance is the difference between the post- and pre-GAENE. We are aware that Barnes et al. (2019) found issues with the GAENE, namely that it may underestimate acceptance as it measures whether respondents would be willing to advocate for evolution. However, at the time this research was taking place, the GAENE was the best available instrument. Additionally, our findings would be on the conservative side if the GAENE underestimates acceptance suggesting that acceptance was even higher among these populations.

To measure student religiosity, we administered, in conjunction with the GAENE, a 15-item religiosity scale that measures religious practice (e.g., church attendance), religious influence (e.g., your religion’s influence on what you eat), and religious hope (e.g., belief in an afterlife) using items on a 6-point Likert scale (Manwaring et al. 2015). Total religiosity was calculated by summing all 15-items for a maximum score of 90. Change in religiosity was calculated by subtracting pre- from post-scores.

### Statistics

To analyze changes in evolution acceptance and religiosity within each course, we ran related-samples Wilcoxon signed-rank tests (the non-parametric equivalent of a paired-samples *t* test) due to the Likert-scale nature of the instruments. We calculated normalized gain scores (using the Average of Gains rule; 40) for both the GAENE and religiosity instruments [(post-score – pre-score)/(Total possible – pre-score)] to illustrate change based on how much room participants had to gain. All assumptions of the models were tested and met.

## Results

### Evolution acceptance

**Fig. 1:**
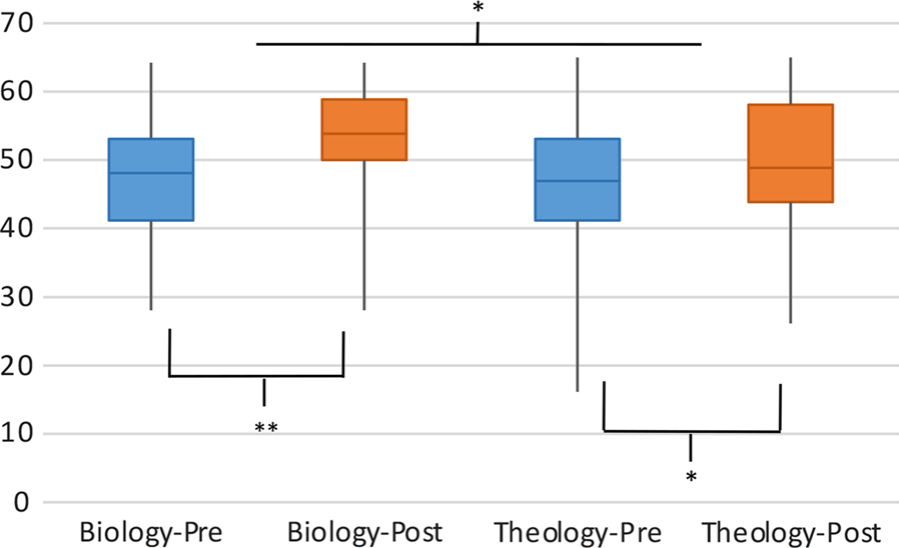
Change in GAENE scores in biology and theology classrooms. The line represents the median, the box encompasses the first to third quartile, and the lines show the minimum and maximum. *p < .05, **p < .001

A Wilcoxon signed-rank test indicated that in the biology class, students experienced a significant increase in evolution acceptance, *M*_pre_ = 72.8%, *M*_post_ = 82.2%, z = 5.05, *p* < .001, with large effect (r = .49); students in the theology class also experienced significant increases, *M*_pre_ = 72.8%, *M*_post_ = 76.7%, z = 3.88, *p* < .001, with medium effect (r = .29) (see Fig. 1).

### Religiosity

**Fig. 2:**
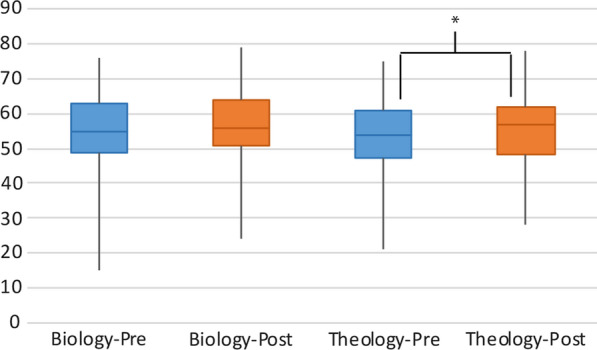
Change in student religiosity in biology and theology classrooms. The line represents the median, the box encompasses the first to third quartile, and the lines show the minimum and maximum. *p < .05

Wilcoxon signed-rank tests showed that neither group of students experienced any decrease in religiosity. Biology students showed no change, z = 1.42, p = .16. Theology students showed a slight increase in religiosity, z = 3.59, p < .001, r = .27 (Fig. 2, Table 2).

**Table 2:** Change in student religiosity

	Pre-mean (%)	Post-mean (%)	Effect (r)	z-statistic	*p*-value
Theology	58.9	60.5	.27	3.59	< .001
Biology	60.9	62.3	.14	1.42	.16

This shows the mean religiosity score from pre- to post-intervention in the Biology and Theology classes, as well as the main effect of each treatment

The Theology class showed a significant, but small increase in religiosity

### Normalized gains

**Fig. 3:**
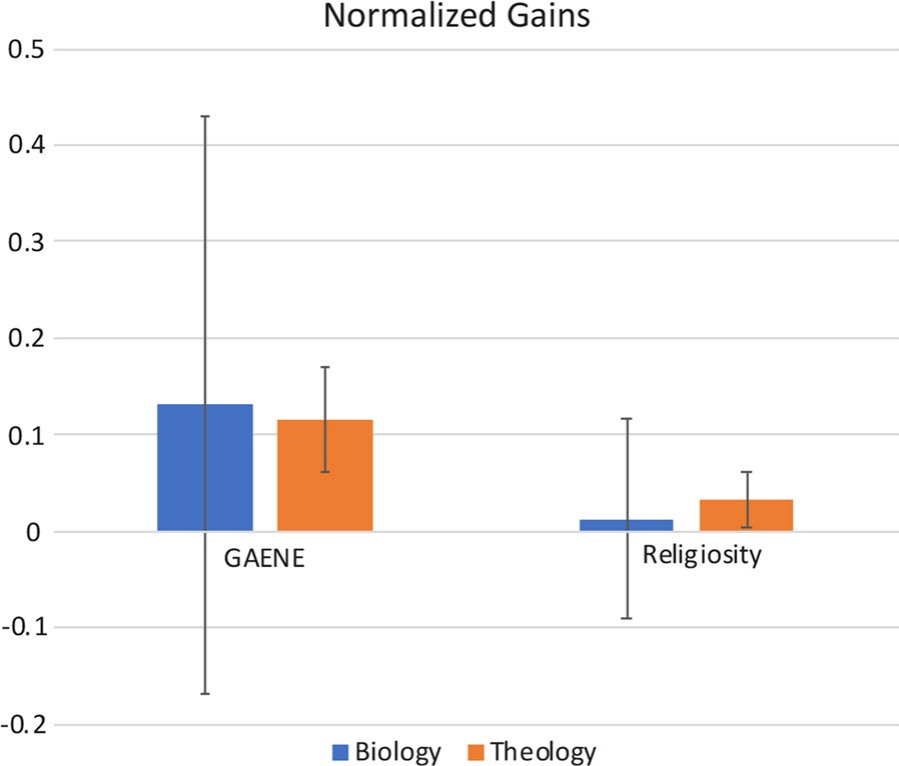
Normalized gains of religiosity and GAENE score in biology and theology classrooms. The line represents the median, the box encompasses the first to third quartile, and the lines show the minimum and maximum

Normalized gains were computed by dividing the difference between pre- and post-test scores by the difference between pre-test scores and the total of the instrument (i.e., how much did they gain given the room they had to gain; see Fig. 3).

## Discussion

We found that both the biology and theology classrooms had significant gains in acceptance of evolution (p < .01, p < .05, respectively). The gains in the biology classroom were significantly greater (p < .05) than in the theology classroom. Neither classroom saw a decrease in religiosity. These results confirm that a reconciliatory model can significantly improve acceptance in both a scientific and religious setting.

As predicted, gains were slightly higher in the biology classroom. Given that these classes are not equivalent in content, instructor, focus of the course, or academic level of students enrolled in the course (e.g., lower or upperclassmen), a direct comparison between these courses is not warranted. However, we can offer several possibilities for why reconciliation may have more of an effect in the biology course in this study. One possibility is the influence of evolutionary content knowledge. As we previously discussed, the literature on the correlation between evolution knowledge and acceptance is mixed. However, while the biology classroom spent a great deal of time teaching the principles and mechanics of evolution, the theology classroom spent essentially no time on evolution content. This seems to support the research (e.g. Dunk et al. 2017) that shows there is a correlation between knowledge and acceptance. In other words, it may be that it takes both knowledge of evolution and a reconciliatory approach to invoke changes in acceptance among Christian students. A second possibility has to do with the nature of science (Dunk et al. 2017; Cofré et al. 2017). As previously cited, teaching students the nature of science leads to significant gains in evolution acceptance. The nature of science, which includes the scientific process and the types of questions science can and cannot answer, was discussed in the biology, but not the theology classroom. Another difference between the biology and theology classrooms was the different role model that each professor presented. Research has shown that a role model who accepts both evolution and religion can lead to gains in acceptance of evolution (Barnes and 2017; Holt et al. 2018). Students could have seen the biology professor as a more authoritative scientific figure, and thus the pathway to reconciliation she presented would have seemed more viable than the one presented by the theology professor. The advancement of students in their respective programs could have also influenced acceptance. The theology class was mostly composed of upperclassmen, most of whom had likely already taken a biology course and every biology course offered at the school teaches evolution (prior experience with biology could also explain why the theology class had higher initial acceptance). The biology class was mainly composed of lowerclassmen who have not had as much exposure to evolution or to the idea that Christianity and evolution could be compatible. Despite these potential differences, the fact remains that these data show a reconciliatory approach to be a potential strategy for increasing student acceptance of evolution and can be effectively used in a scientific or religious course setting.

### Religiosity

It is important to note that significant change in evolution acceptance among students is not accompanied by significant decreases in religiosity. Students do not appear to be losing a part of their religious identity or changing their practices because they are accepting evolution. This dispels a potential fear among religious communities that the acceptance of evolution will pull religious individuals away from their faith.

## Limitations

Although our results are statistically significant, we realize that there are limitations in our study design. First, it has been well documented in the literature that different results can be obtained using different instruments of acceptance (Barnes et al. 2019). We acknowledge that the GAENE has been criticized for not dividing microevolution, macroevolution, and human evolution, leading to students potentially using different definitions when answering the question; in addition, it has been criticized for having advocacy built into the question and therefore may underestimate evolution acceptance. Thus, a note of caution should be given that results of our intervention may vary depending (Barnes et al. 2019) on instrumentation used to assess evolution acceptance.

A second limitation is in the design of the courses. In an effort to use as naturalistic an environment for these interventions as possible, courses were not tightly controlled for equivalent student enrollment (i.e., the theology class is normally taken by upperclassmen; the biology class is a part of the typical freshman series). By comparing pre- to post-acceptance, we attempted to control for any potential differences in prior exposure. However, other factors could certainly play a role in a student’s willingness to change their views on evolution based on their previous experiences as an undergraduate. In addition, the courses were intendedly different in their content and emphases, one being religiously oriented and the other a biology class. We simply sought to show that reconciliation can be effective in various environments. However, any direct comparison between the two courses would be inappropriate.

We also acknowledge limitations in applying the reconciliatory model to broader populations. We recognize many biology professors may not be religious, and many theologians may not have a solid understanding of evolutionary science, limiting the ability of biology and theology professors to be effective role models. However, there are many resources professors can use with students (for example, the Smithsonian’s Human Origins Broader Social Impacts Committee provides videos from scientists of diverse religious backgrounds to help students navigate faith and science (Smithsonian’s Broader Social Impacts Committee 2009). We also encourage all professors to employ Religious Cultural Competence in Evolution Education (ReCCEE; (Barnes and Brownell 2017)) to assist in their reconciliatory model. The professors who taught these classes employed ReCCEE practices by having frank, respectful discussions about theological beliefs and evolution and their potential compatibility. Both professors offered students different perspectives through which students could hold evolution within their faith traditions, instead of dropping their beliefs completely in order to accept evolutionary theory.

Another limitation of this study is that we did not measure knowledge of evolution nor knowledge of the nature of science. Further research is needed to explore how these variables factor into acceptance in both scientific and theological classrooms.

## Conclusions

This study confirms prior research (Lindsay et al. 2019) that a reconciliatory approach is an effective way of helping students come to accept evolution. Although the gains in evolution acceptance in the biology classroom were significantly higher than the gains in the theology classroom, this research shows that a theology classroom could be a viable setting for increasing student acceptance of evolutionary theory. It is possible that if students in a theology classroom were encouraged to learn basic principles of evolution on their own, it could lead to even greater gains in the acceptance of evolutionary theory. We also establish that gains in evolution acceptance can be established without a decrease in religiosity.

Scientists have been warning of the dangers of public rejection of science for decades (Augustine 1998; Bishop 1989; Pasek 2018), yet rejection of science is still rampant and a great threat to our society. The success of the use of the reconciliatory approach shows a simple way that the denial of evolutionary theory could be overcome. Importantly, our results show that efforts to increase the acceptance of evolutionary theory can take place in venues outside of a traditional biology classroom. Additionally, the success of the reconciliatory approach could potentially be used to increase the acceptance of climate science, vaccines, GMOs, etc. We call on educators to implement reconciliatory approaches where possible, to confront scientific denialism.

## Data Availability

All data is available in BYU Scholars Archive (https://scholarsarchive.byu.edu#1024).

## Footnotes

1 Wesleyanism is a movement within Protestant Christianity tracing its roots back to the 18^th^Century Evangelical Revival in England, in part led by John and Charles Wesley, and includes denominations such as the United Methodist Church and the Church of the Nazarene. Wesleyan Christians generally reject a fundamentalist approach to scripture that asserts the historical and scientific inerrancy of scripture and therefore necessitates conflict between biblical faith and contemporary science as found in the Young Earth Creation movement. Wesleyans consider scripture to have little or no concern with the subjects and methods of modern science and history and therefore instead focus on scripture as the authoritative guide for Christian salvation and life. .

2 This aligns well with the official Nazarene article of faith on scripture which states that biblical ‘inerrancy’ is limited to ‘revealing God’s will for us concerning *all things necessary for our salvation*.’.
